# Acute Infectious Gastroenteritis: The Causative Agents, Omics-Based Detection of Antigens and Novel Biomarkers

**DOI:** 10.3390/children8121112

**Published:** 2021-12-02

**Authors:** Haziqah Hasan, Nor Ashika Nasirudeen, Muhammad Alif Farhan Ruzlan, Muhammad Aiman Mohd Jamil, Noor Akmal Shareela Ismail, Asrul Abdul Wahab, Adli Ali

**Affiliations:** 1 Department of Pediatric, Faculty of Medicine, Universiti Kebangsaan Malaysia, Jalan Yaacob Latif, Kuala Lumpur 56000, Malaysia; a175099@siswa.ukm.edu.my (H.H.); a176982@siswa.ukm.edu.my (N.A.N.); a176986@siswa.ukm.edu.my (M.A.F.R.); a177121@siswa.ukm.edu.my (M.A.M.J.); 2 Department of Biochemistry, Faculty of Medicine, Universiti Kebangsaan Malaysia, Jalan Yaacob Latif, Kuala Lumpur 56000, Malaysia; nasismail@ukm.edu.my; 3 Department of Medical Microbiology and Immunology, Faculty of Medicine, Universiti Kebangsaan Malaysia, Jalan Yaacob Latif, Kuala Lumpur 56000, Malaysia; saw@ppukm.ukm.edu.my

**Keywords:** acute infectious gastroenteritis, aetiological agents, biomarkers, omics, genotypes

## Abstract

Acute infectious gastroenteritis (AGE) is among the leading causes of mortality in children less than 5 years of age worldwide. There are many causative agents that lead to this infection, with rotavirus being the commonest pathogen in the past decade. However, this trend is now being progressively replaced by another agent, which is the norovirus. Apart from the viruses, bacteria such as *Salmonella* and *Escherichia coli* and parasites such as *Entamoeba histolytica* also contribute to AGE. These agents can be recognised by their respective biological markers, which are mainly the specific antigens or genes to determine the causative pathogen. In conjunction to that, omics technologies are currently providing crucial insights into the diagnosis of acute infectious gastroenteritis at the molecular level. Recent advancement in omics technologies could be an important tool to further elucidate the potential causative agents for AGE. This review will explore the current available biomarkers and antigens available for the diagnosis and management of the different causative agents of AGE. Despite the high-priced multi-omics approaches, the idea for utilization of these technologies is to allow more robust discovery of novel antigens and biomarkers related to management AGE, which eventually can be developed using easier and cheaper detection methods for future clinical setting. Thus, prediction of prognosis, virulence and drug susceptibility for active infections can be obtained. Case management, risk prediction for hospital-acquired infections, outbreak detection, and antimicrobial accountability are aimed for further improvement by integrating these capabilities into a new clinical workflow.

## 1. Introduction

AGE is defined as a diarrhoeal disease of rapid onset presenting with the incidence of three or more soft or liquid stools, or three bouts of vomiting per 24 h, with addition of abdominal pain, or fever [1,2]. This is due to the damage at the villous brush border of the intestine, which reduces the capacity to absorb intestinal contents and subsequently results in osmotic diarrhoea. Some of the causative agents would also release their toxins which bind to the specific enterocyte receptors, releasing chloride ions into the intestinal lumen which eventually causes secretory diarrhoea [3,4].

Acute infectious gastroenteritis (AGE) often affects children aged below 5 years old and causes the second highest mortality rate globally after pneumonia [5,6]. The condition is more common in developing countries than in developed countries. There have been estimated to be about 125 million gastroenteritis cases among infants 0–11 months of age and 450 million cases in children 1–4 years of age, prominently in developing countries [7]. AGE undeniably contributes to social and emotional impacts either to the parents or children, together with financial burdens. Extra expenditure is required to purchase diapers, doctor’s consultation, and medications, which some found financially bothersome [8]. Additionally, children will have to endure embarrassment and fright, whereas parents might suffer from fatigue and irritability during that period [9].

Considering that each causative pathogen of AGE has a different course of diseases and complications, different management and prevention strategies are required. Thus, identifying the correct causative pathogen, especially differentiating if it is either bacterial or viral in origin, and also predicting which patient likely requires more extensive treatment, will help with the management plan and reduction in comorbidities. Thus, this review will explore the current available biomarkers and antigens available for the diagnosis and management of the different causative agents of AGE, and subsequently, intends to look at the application of omics technologies in elucidating and discovering various infectious agents and novel biomarkers related in the management of AGE.

## 2. Causative Agents of AGE

Viruses, bacteria, and parasites are among the most common infectious agents causing AGE in children. Amongst them, viruses such as Rotavirus and Norovirus contribute to the greatest rate of infection. In addition to that, astrovirus, adenovirus, and bacteria (*Salmonella* and *Eschrichia coli*) as well as parasites (*Entamoeba histolytica*) are also equally prominent. These agents are transmitted to the host via various routes such as faeco–oral, person-to-person, and by fomites. Table 1 represents the causative agents from the highest prevalent to the less common (virus > bacteria > parasite) and their current methods of detection.

### 2.1. Rotavirus

Rotavirus is the most common agent causing AGE in children worldwide [10,11,12,13,14,15]. It has been suggested to be one of the most pathogenic causative agents in AGE which correlates with infectious diarrhoea, vomiting, and higher frequency of fever [13,16,17,18]. It was mostly reported among children aged 0–14 years old, with the highest incidence among children less than 5 years [14,15,16,19,20,21]. The prevalence was highly reported during the dry season [15,21], bottle-fed children [15,22,23], and children with group A blood type [15,24,25]. Moreover, laboratory investigations revealed a significantly high level of serum transaminase among children infected with rotavirus [26,27,28]. Among many studies conducted worldwide, it was found that rotavirus infected children have a high chance of developing dehydration as a complication, and thus, must be closely monitored [7,16,29,30].

### 2.2. Norovirus

Norovirus is an emerging cause of acute gastroenteritis, responsible for approximately 17–18% of all acute gastroenteritis cases worldwide, especially among developed countries [31,32,33,34,35]. The age group under 1 year old has the highest frequency of norovirus infection with additional risk factors which include male sex [34]. Norovirus is detected throughout the year, with the autumn and winter seasons observed to report a higher frequency of cases [31,36,37]; the detection rate of norovirus cases correlates positively with humidity [37]. Co-infection with rotavirus [38,39], astrovirus [40], and *Salmonella* [39] may occur in certain cases. Norovirus infections are observed to cause more severe symptoms of gastroenteritis in children compared with rotavirus, especially after the introduction of the rotavirus vaccination period [41]. Individuals with AB blood group have less susceptibility towards GII.4 noroviruses, whereas those with the O blood group are more susceptible to GI.4, GII.4, GII.17, and GII.18-Nica viruses [42]. GII.4 is the most common norovirus genotype causing infection in children [34,43,44]. However, recent epidemiological data observed that GII.2[P16] is currently an emerging norovirus strain in East Asia and Europe [45]. Norovirus-infected patients are more likely to have a longer duration of infection and more frequent vomiting in a day, but less likely to report fever in comparison with other causes of infective gastroenteritis [46].

### 2.3. Astrovirus

With an astrovirus-causing acute gastroenteritis’ global average incidence of 11%, the highest prevalence of human astrovirus (HAstV) infections was in the group of children between 37 and 48 months old [47,48]. HAstV infection mainly occurs during the dry season in the African continent; meanwhile, the highest occurrence reported in the tropical areas is often in the rainy season and winter season in temperate climate countries [47,49]. Patients usually manifested with diarrhoea, fever, vomiting, and abdominal pain [48,50].

### 2.4. Enteric Adenovirus Serotypes 40 and 41

Between 2.3 and 5% of the diarrhoea cases in children were caused by adenoviruses (AdV) serotypes 40 and 41 [51]. Enteric adenovirus serotypes 40 and 41 account for both sporadic or epidemic gastroenteritis in infants and young children, which are detected throughout the year with a summer peak between May and July [51,52,53]. Those infected with this pathogen will acquire mild fever and vomiting, abdominal pain, bloody diarrhoea, respiratory symptoms such as cough, and/or secondary lactose malabsorption [53,54]. Patients with adenovirus gastroenteritis have a significantly higher CRP (mean 3.4 mg/dL) and ESR value (mean 24 mm/h) compared with other gastroenteritis pathogens, and pulmonary-associated symptoms and vomiting frequency are increased in patients with this infective gastroenteritis [55]. Intravenous cytosine nucleotide analogue (CDV) that inhibits DNA polymerase has the highest in vitro activity against adenovirus, and is the preferred therapeutic agent. Elevation in lymphocyte counts (specifically CD4) were correlated with the clearance of AdV infection and improvement in survival [56,57].

### 2.5. Salmonella

*Salmonella* has become a major foodborne pathogen across the globe, causing about 3.4 million cases and 681,316 annual deaths, with 63.7% of cases occurring in children under 5 years of age [58,59,60]. There were at least 150 non-typhoidal *Salmonella* serotypes that can cause gastroenteritis, with *Salmonella* Typhimurium and *Salmonella* Enteritidis being the most common serotypes [59,61,62]. A study in Greece recorded that the highest rate of infection was in August, with infants being the most vulnerable group [63]. The contributing factor of salmonellosis was mainly related to consumption of contaminated food and poor clean water supply [64,65]. Salmonellosis can cause the increase in C-reactive protein values (CRP), erythrocyte sedimentation rate (ESR) and body temperature [66]. In terms of management, the recommended empiric parenteral therapy includes cefotaxime or ceftriaxone, whereas oral therapy includes amoxicillin, trimethoprim-sulfamethoxazole, or azithromycin. *Salmonella* isolates have a high resistance towards at least one antimicrobial agent [61,67], especially towards clindamycin, oxacillin, penicillin, and vancomycin, thus antibiotic susceptibilities of *Salmonella* must be determined for the targeted antibiotic therapy [62].

### 2.6. Escherichia coli

The WHO Global Burden of Foodborne Diseases report estimates that more than 300 million illnesses and nearly 200,000 deaths are caused by diarrheagenic *Escherichia coli* (DEC) globally each year. DEC is one of the major causal agents of diarrhoea in children under 5 years of age in developing countries [68,69,70], whereas children under 2 years of age are at the highest risk of infection with Enteropathogenic *Escherichia coli* [71,72] and *Escherichia coli* O157:H7 [71,73]. However, the major cause of paediatric infections in certain areas such as Ahvaz, Iran, were non-O157:H7 *E. coli* [74]. The incidence of non-O157 Shiga Toxin-producing *Escherichia coli* has been increasing in recent years, including those caused by serotypes O26, O45, O103, O111, O121, and O145 [75]. Co-infections can occur especially between EPEC and *Campylobacter* spp. [76]. Interestingly, Enteropathogenic *Escherichia coli* cases are less frequently detected in Malaysia and its similar geographical/climatic areas, in comparison with a country such as Iran where it has been reported that acute gastroenteritis was greatly caused by this strain [77]. Most DEC are sensitive to ciprofloxacin and the empirical antibiotic of choice [78,79].

### 2.7. Entamoeba histolytica

Other than viruses and bacteria, parasites such as *Entamoeba histolytica* also play a role in causing acute gastroenteritis in children [80,81,82]. Children infected with *Entamoeba histolytica* were mostly presented with tenesmus [81,82,83], fever [81,82], vomiting [81,84], abdominal cramps [81,83,84], and bloody diarrhoea [8,82,83]. Through laboratory investigations, it was found that infection with *Entamoeba histolytica* results in leukocytosis [80,81,84,85], elevation of CRP [74,78,79,80,84,85], elevation of ESR [81], elevation of serum alkaline phosphatase, and serum transaminase levels [81,86]. Eating unwashed or raw vegetables [86,87,88,89] and poor water hygiene [82,90,91] were identified to increase the risk of *Entamoeba histolytica* infection.

## 3. Biomarkers in the Detection of Common Aetiological Agents of AGE

There are various biomarkers utilised in the detection of aetiological agents of AGE via various platforms, with the most common being the detection of antigens via enzyme immunoassay (EIA), enzyme-linked immunosorbent assay (ELISA), and immunochromatography. Moreover, detection of genes could also be conducted via procedures such as real-time PCR (RT-PCR) and multiplex real-time PCR. These are cost-effective methods that can be implemented in the future for detection of biomarkers identified via omics technologies. The breadth of the available tests, their sensitivity, and specificity are summarized in Table 1.

### 3.1. Rotavirus

As the most common inflicted pathogen causing AGE in children, several biomarkers have been developed and available commercially for the detection of rotavirus. The widely available biomarker platform utilized is the enzyme immunoassay (EIA) to screen for the rotavirus antigen [10,11,13,15,19,22,26,92,93,94]. Amongst EIA kit used were Premier Rotaclone, Meridian Bioscience Inc., Cincinnati, OH, USA [10,11,12,19,93,94], RIDASCREEN Rotavirus R Biopharm AG, Darmstadt, Germany [15,93], and ProSpect Rotavirus Test, Oxoid Ltd., UK [22,93]. Rotavirus antigens can also be detected by using enzyme-linked immunosorbent assay (ELISA) [12,16,18,20,28,30] which includes ELISA kits such as Premier Rotaclone, Meridian Bioscience, Inc. [12], Fecal Rotavirus Antigen ELISA Kit (EDI, CA, USA) [16], ProSpecTM Rotavirus Microplate Assay, Oxoid [20] and Rota Antigen Test Device, Cambridge [28]. ELISA can also be used in the detection of rotavirus-specific IgM [29]. Other methods in detection of rotavirus antigen were immunochromatography [25,27] and latex agglutination [23,92,95]. In addition, polyacrylamide gel electrophoresis (PAGE) was carried out to determine the electropherotype of rotavirus strains [10,19,92]. Moreover, samples that were rotavirus positive for ELISA or EIA were sent for genotyping using reverse-transcription polymerase chain reaction (RT-PCR) to determine whether they belong to particular G/P genotypes [11,16,19,20,25,29,96,97]. A multiplex real-time RT-PCR is an advanced approach for a high-throughput rotavirus genotype characterization for monitoring circulating rotavirus wild-type strains, which is more robust in identifying a novel strain [98].

### 3.2. Norovirus

Viral RNA and antigens are the main biomarkers for detection of norovirus infection. Real-time quantitative polymerase chain reaction, Multiplex Gastrointestinal Platforms, enzyme immunoassays (EIAs) and genotyping are the main methods used in laboratory diagnosis of norovirus [99]. Cepheid Xpert^®^ Norovirus kit automates sample processing, nucleic acid extraction, and real-time reverse transcription polymerase chain reactions (RT-PCRs) for detection and differentiation of norovirus GI and GII, which account for the majority of norovirus infections worldwide [100,101]. Another real-time PCR platform, RIDA^®^GENE Norovirus, can also be used as an alternative in detecting the virus [102]. Although PCRs are highly sensitive and specific, they are expensive and require specialized techniques and equipment. Rapid diagnostic tests are usually carried out during outbreak screening and patient management, which include immunochromatographic test, enzyme immunoassay (EIA), enzyme-linked immunosorbent assay (ELISA) and fluorescence immunoassay (FIA). RIDA^®^QUICK Norovirus (R-Biopharm AG, Darmstadt, Germany) is one of the most used rapid immunochromatographic tests to detect norovirus [103,104,105]. Other immunochromatographic tests that are still used include QuickNaviTM—Norovirus 2 [106,107]. RIDASCREEN^®^ Norovirus 3rd Generation is an example of ELISA that is still currently in use [108,109]. For FIA, the Automated Fluorescent Immunoassay System NORO (AFIAS-Noro) assays (Boditech Med Inc, Gangwon-do, South Korea) are newly developed diagnostic tests for norovirus infections [95].

### 3.3. Astrovirus

Stool samples can be investigated using RT-PCR to detect the presence of human astrovirus (HAstV) [47,48,55]. One of the available kits is the RT-PCR Luminex Assay, with which a portion of the ORF2 capsid region is targeted by using a set of specific reverse primers labeled with biotinTEG at 5′-ends and specific probes sequences [110]. A one-step, accelerated, real-time RT-LAMP (rRTLAMP) assay can also be used by targeting the 5′-end of the capsid gene for rapid and quantitative detection of HAstV [111,112].

### 3.4. Enteric Adenovirus Serotypes 40 and 41

Adenovirus antigen and hexon-coding gene in enteric adenovirus serotypes 40 and 41 can be recognised and used for screening and detection methods of this pathogenic agent infection. BioNexia RotaAdeno and RIDA Quick Rota-Adeno-Combi R-Biopharm, which are the immunochromatographic tests (ICT) and LIAISON Adenovirus chemiluminescence immunoassays (CLIA) can be utilised to detect enteric adenovirus antigen [113,114]. The samples from ICT and LIAISON CLIA can subsequently undergo RT-PCR for genotyping of hexon-coding genes by using a specific TaqMan Array Card, which is a 384-well singleplex real-time PCR format that has been recognised to detect multiple infection targets [115,116].

### 3.5. Salmonella

As for *Salmonella* infection, the virulence genes include flagellin (*filcC*), *invA*, *invF*, *sitC*, *hilAgene*, *sipC*, *sipF* genes as well as heat stable enterotoxin gene, *parE* [117]. Multiplex real-time PCR such as RIDA GENE-gastrointestinal kits, EntericBio real-time Gastro Panel I, and Seeplex Diarrhea ACE detection has allowed a more rapid detection of multiple targets in a short period of time and some of the tests was followed by hybridisation to microarray/macroarray to achieve multiparametric detection of AGE aetiological agents [118]. In terms of sensitivity and specificity, RIDA GENE-gastrointestinal kits were reported to have 25% sensitivity and 99.7% specificity [119,120,121]. As for EntericBio real-time Gastro Panel I, its sensitivity and specificity were much higher, which were 100% and 97.8%, respectively [120]. Seeplex Diarrhea ACE has a sensitivity of 40–100% and specificity of 96–100% [119,122,123].

### 3.6. Escherichia coli

Most of the current omics approaches were focused on the detection of Shiga Toxin-producing *Escherichia coli* (STEC) infections. This is due to the fact that accurate diagnosis of STEC infection is very crucial because appropriate early treatment decreases the risk of serious complications and improves overall patient outcome, especially in children [124,125]. Although non-O157 serotypes account for the majority of STEC infections, they are significantly under-reported because frontline microbiology laboratories mainly focus on the detection of O157 STEC using specific agar-based methods [126]. CHROMagar STEC is a new chromogenic medium invented to improve detection of STEC, which detects O157 and non-O157 STEC through a chromogenic substrate [126,127]. However, PCR is a more sensitive test than culture [126,127]. RIDA^®^GENE real-time PCR kits EAEC, EHEC/EPEC, and ETEC/EIEC (R-Biopharm, Darmstadt, Germany) all can be used to detect the *aatA* and *aggR*, *eae*, and *elt* and *estA* genes of Enteroaggregative, Enteropathogenic, and Enterotoxigenic *Escherichia coli*, respectively [128]. These will ensure that different pathotypes of diarrheagenic *Escherichia coli* can be detected and differentiated successfully.

### 3.7. Entamoeba histolytica

*Entamoeba histolytica* infection can cause a significant decrease in serum leptin and has been suggested to be a potential biomarker for this pathogenic infection [129], which can be detected using the ELISA technique (kit supplied by RayBiotech.Inc, Guangzhou, China). Moreover, there was also a marked increase in HDL, obestatin, calprotectin and SIgA concentration level with a concurrent decrease in cholesterol, triglyceride, LDL and VLDL concentration levels. All of these serums can be analysed and measured using ELISA techniques [130]. ELISA (Techlab II *Entamoeba histolytica*) can also be used to detect *Entamoeba histolytica* antigen presented in stool with a specificity of 100% but with a low sensitivity of 19.2% [131]. Thus, multiplex PCR is a more favourable option as it has sensitivity of 100% with specificity of 95.8% [132]. During progression of amoebiasis, a small non-coding RNA known as microRNA (miRNA) is involved in promoting apoptosis in epithelial colon cells and comprehensive profiling of miRNA using Taqman Low-Density Arrays showed a significant interaction between miRNA and parasite presented [133]. Taqman Low-Density Arrays has a sensitivity of 92% and a specificity of 100% in diagnosis of *Entamoeba histolytica* infection [134].

**Table 1:** Biomarkers of aetiological agents and detection methods.

Agent	Biomarker	Detection Method	Brand	Sensitivity	Specificity	References
Rotavirus	Rotavirus antigen	ELISA	Rota Antigen Test Device, Cambridge	86–98%	92–96%	[28]
		EIA	Premier Rotaclone, Meridian Bioscience Inc., Cincinnati, OH, USA	76.8–77.8%	100%	[93,94]
			RIDASCREEN^®^ Rotavirus	82.1–97.8%	99.1–100%	[93]
			ProSpect Rotavirus Test, Oxoid Ltd., UK	75%	100%	[93]
	Rotavirus RNA	RT-PCR	Primerdesign Genesig^®^ Kit	100%	100%	[97]
		Real-time RT-PCR	GeneAmp EZ rTth RNA PCR kit (Applied Biosystems, Inc., Foster City, CA, USA)	98.8–100%	99.7–100%	[98]
Norovirus	Norovirus antigen	ICT	RIDA^®^QUICK Norovirus N1402	72.8–87%	97–99.5%	[117,118]
			QuickNaviTM Norovirus 2	27.5%	97.7%	[120]
		ELISA	RIDASCREEN^®^ Norovirus 3rd Generation	84.6–85.7%	>96%	[121,122]
		FIA	AFIAS-Noro	66%	97.6%	[95]
	Norovirus RNA	Real-time RT-PCR	Cepheid Xpert^®^ Norovirus	100%	100%	[114]
		Real-time PCR	RIDA^®^GENE Norovirus	98%	98%	[102]
Astrovirus	*ORF2* gene	RT-PCR	RT-PCR Luminex Assay	100%	100%	[123]
			rRTLAMP Assay	94%	100%	[124,125]
Enteric Adenovirus Serotypes 40 and 41	Adenovirus antigen	ICT	BioNexia RotaAdeno	60%	98.8%	[126]
			RIDA Quick Rota-Adeno-Combi R-Biopharm AG	72.7%	98.2%	[127]
		CLIA	LIAISON Adenovirus	77%	98.8%	[126]
	Hexon-coding gene	RT-PCR	TaqMan Array Card	100%	100%	[129]
*Salmonella*	*Salmonella* virulence genes	Multiplex PCR	RIDA^®^GENE-gastrointestinal kits	25%	99.7%	[103,104,105]
			EntericBio real-time Gastro Panel I	100%	97.8%	[104]
			Seeplex Diarrhea ACE	40–100%	96–100%	[103,106,107]
Shiga Toxin-producing *Escherichia coli* (STEC)	Shiga toxins (stx) serotypes	Multiplex PCR	Seeplex Diarrhea ACE	100%	99.6–100%	[110]
		Real-time PCR	TaqMan™ STEC	100%	100%	
		Culture medium	CHROMagar STEC	84.6–85.7%	87–95.8%	
Enteropathogenic *Escherichia coli* (EPEC)	*eae* gene	Real-time PCR	RIDA^®^GENE EHEC/EPEC	84%	97%	[112]
Enterotoxigenic *Escherichia coli* (ETEC)	*elt* and *estA* genes		RIDA^®^GENE ETEC/EIEC	83%	100%	
Enteroaggregative *Escherichia coli* (EAEC)	*aatA* and *aggR* genes		RIDA^®^GENE EAEC	69%	100%	
*Entamoeba histolytica*	*Entamoeba histolytica* antigen	ELISA	Techlab II *Entamoeba histolytica*	19.2%	100%	[131,132,133]
		Multiplex PCR	Performed on Mx3005P detection system	100%	95.8%	[134]
	microRNA (miRNA)	RT-PCR	Taqman Low-Density Arrays	92%	100%	[135]

## 4. Omics-Based Technologies for AGE Diagnosis and Management

Recent advancement in omics technologies has enabled researchers to further elucidate the potential causative agents for AGE, as only known pathogens can be detected and identified by the current molecular target-dependent techniques. Metagenomics based on next-generation sequencing (NGS) platform, is a target-independent assay that allows concurrent detection and genomic characterization of all microorganisms present in a sample [136]. The latest NGS platform was first applied in AGE studies to look at the most prevalent genotype in rotavirus. The method was conducted on rotavirus positive specimens to further elucidate the most prevalent genotype in various geographical areas [135,137,138,139,140]. Metagenomic approach using NGS can also be used to analyze norovirus strain diversity [141,142,143]. Additionally, next generation whole genome sequencing performed on fecal samples is proposed as a powerful tool for the diagnosis and genetic analysis of norovirus infection [144,145].

Similarly, astrovirus infection can be detected using the NGS platform via metagenomic analysis [136,146]. Amino acid sequence of the capsid region (ORF2) classified the identified HAstV infection into the “classical” genotypes (Mamastrovirus 1 (MAstV-1) species) or the “novel” recombinant genotypes (MAstV-6, 8, and 9 species) [147]. The other astrovirus genotypes that can be detected using NGS are also listed in Table 2 [146]. By using the similar approach, enteric adenovirus serotypes 40 and 41 can be specifically detected, in addition it is able to correctly identify and differentiate the different genotypes of human adenovirus (HAdV) A–G [136,148]. Furthermore, *Entamoeba histolytica* has also been analysed for its respective strains using NGS platform [149] (Table 2).

Single-cell transcriptomics is the latest omics approach to unravel the dynamics of cellular reconstruction by measuring the concentration of mRNA to thousands of genes in each cell, which can be applied in the study of gastrointestinal pathogens [150]. Transcriptomics analysis using Serial Analysis and Cap Analysis of Gene Expression (SAGE/CAGE), and microarrays also have been used to identify the present of nadA in detection of *Salmonella enterica* [151,152,153] and EHEC EDL933 in detection of *Escherichia coli* [154].

Proteomics is specially dedicated to protein analysis, by looking at the different composition of amino acids. This platform has been growing steadily and widely utilized with the supplementation of advances in both mass spectrometry and bioinformatics [155]. In detection of *Salmonella* Enteritidis, proteomic approaches have been used to detect isogenic Type Three Secretion System-I (TTSS-I) known as M1511 and its wild-type isolate, P125109 using a high-resolution Fourier transform mass spectrometer [156]. For *Escherichia coli* identification, matrix-assisted laser desorption ionization time-of-flight mass spectrometry (MALDI-ToF MS) can also be used because it is a rapid, low cost, reliable and accurate proteomic method for *E. coli* identification up to the subspecies level compared with classical culture microbiological techniques [157,158].

Metabolomics belongs to the ‘omics’ family that deals with the characterization of the set of metabolites in a given biological system [159]. As such, metabolomics analysis can be used to detect *Salmonella enterica* serovar Typhimurium. The most widely used techniques in metabolomics analysis are gas chromatography-mass spectrometry (GC-MS), liquid chromatography–mass spectrometry (LC-MS), and capillary electrophoresis coupled to mass spectrometry (CE-MS) [154,160,161]. Liquid chromatography–mass spectrometry (LC-MS) is also the metabolomic analysis technique used to detect changes in gut microbiota in children with diarrhoea caused by diarrhoeagenic *Escherichia coli* (DEC), as DEC-infected children and healthy children have a statistically different composition of microbiota [162].

**Table 2:** Omics Technologies for Elucidation of AGE Causative Agents.

Omics	Platform	Infectious Agents Detected	Potential Biomarkers	References
Metagenomics	Next Generation Sequencing	Rotavirus	G1P[8]	[135]
			G9P[8]	[135,138]
			G3P[8]	[137]
			G3P[6]	[137]
			G8P[8]	[139,140]
		Norovirus	GI genotypes GII genotypes	[135,144,145]
			GII.4	[137]
		Astrovirus	MAstV-1 MAstV-6 MAstV-8 MAstV-9	[142]
			StAstV Bovine AstV Deer AstV Dromedary AstV Porcine AstV Murine AstV Wild boar AstV Rabbit AstV Rat AstV	[141]
		Adenovirus	HAdV A HAdV B HAdV C HAdV D HAdV E HAdV F HAdV G	[130]
		*E. histolytica*	KU27 KU50	[144]
Transcriptomics	Serial Analysis and Cap Analysis of Gene expression (SAGE/CAGE) Microarray	*Salmonella enterica*	nadA gene	[155,156,157]
		Enterohaemorrhagic *Escherichia coli*	EHEC EDL933	[146,147,148]
Proteomics	Fourier transform mass spectrometer	*Salmonella enteritidis*	TTSS-I (M1511) P125109	[150]
	Matrix-assisted laser desorption ionization time-of-flight mass spectrometry (MALDI-ToF MS)	*Escherichia coli*	Mass peaks ranged from approximately 3000 to 15,000 m/z for different strains of *E.coli* (EPEC, EIEC and DAEC strains).	[151]
Metabolomics	Gas chromatography-mass spectrometry (GC-MS) Liquid chromatography–mass spectrometry (LC-MS) Capillary electrophoresis coupled to mass spectrometry (CE-MS)	*Salmonella enterica* Serovar Typhimurium	22 metabolites were identified in greater abundance and these metabolites triggered oxidative stress.	[148,154,155]
	Liquid chromatography–mass spectrometry (LC-MS)	Diarrheagenic *Escherichia coli* (DEC)	Higher levels of histamine and lower levels of ornithine in DEC samples than in the healthy group.	[156]

Emerging omics technologies have evoked the elucidation of significant infectious agents of AGE. These multi-omics approaches can be used as the basis for the detection of the biomarkers which can be elucidated in the future through much cheaper methods such as PCR and ELISA in the clinical setting. Therefore, the technology in deciphering the role of causative agents of AGE should be explored and made accessible. Since multidrug resistance and healthcare-associated infections pose threats to current epidemic, this demand revolution in management strategies, hence multi-omics approaches can be one of the potential solutions in detecting potential biomarkers. Ability to manage infections can be transformed with routine access of pathogen genomic data, but only if we can amalgamate with other data for critical outcomes. Extensive clinical data related to pathogen genotypes in the long term will enable prediction of prognosis, virulence, and drug susceptibility for active infections. Integrating these capabilities into a new clinical approach in detecting biomarkers should improve case management, risk prediction, outbreak detection, and antimicrobial accountability of AGE.

## 5. Conclusions

Although rotavirus remains the main pathogen causing acute gastroenteritis in children, other pathogenic agents such as norovirus is evolving to emerge as one of the important AGE-causative agents and requires attention in the future studies. This is without ignoring the fact that bacteria and parasite pathogens also have a continuous and very relevant role in causing acute infectious gastroenteritis especially among children. With the emergence of other causative pathogens, different numerous mutations resulted in more pathogenic strains, as well as lack of reliable prognostic parameters, more robust diagnostic and predictive biomarkers need to be considered. Assuming the cost as the barrier, NGS and other omics-based approaches can be used as the fundamental methodologies for novel biomarker detection in clinical microbiology. With the wider and hopefully in the near future more accessible utilization of different omics-based approaches, these modalities should be readily considered and integrated for a more holistic, and comprehensive management of patients with AGE.

## Data Availability

Not applicable.
